# 776. Clinical Practice Gap Analysis of Physicians’ Understanding of Cytomegalovirus

**DOI:** 10.1093/ofid/ofad500.837

**Published:** 2023-11-27

**Authors:** Amy Larkin, Arun S Nair, Iwona Misiuta

**Affiliations:** Medscape Education, Nicholasville, Kentucky; Medscape Education, Nicholasville, Kentucky; Medscape Education, Nicholasville, Kentucky

## Abstract

**Background:**

Understanding clinical practice related to awareness and clinical understanding of cytomegalovirus (CMV) and can inform development of tools to improve physician practices and patient screening, diagnosis and management.

**Methods:**

A survey instrument of 25 multiple choice, knowledge- and case-based questions allowed participants to assess their clinical knowledge with regard to CMV. These questions are designed to assess clinicians’ baseline knowledge, competence, and current practice. Immediate peer-response data solidify support for correct responses. The survey was available online to physicians without monetary compensation or charge. Respondent confidentiality was maintained and responses were de-identified and aggregated prior to analyses. Initial data collection occurred from July 27, 2022 to October 18, 2022.

**Results:**

In total, 107 OB/GYNs and 77 pediatricians completed the full assessment within the data collection period. Physicians demonstrated gaps in the following areas:

**Conclusion:**

This educational research on assessment of physicians’ clinical practices yielded important insights into clinical gaps related to CMV among OB/GYNs and pediatricians. While the gaps were similar in most areas, OB/GYNs showed larger gaps in identifying source of transmission, and management of possible congenital CMV compared with pediatricians, whereas pediatricians showed larger gaps in identifying seropositivity rates and diagnosing CMV.

**Disclosures:**

**All Authors**: No reported disclosures

**Table:** 

Topic	Incorrect Responses to Knowledge and Clinical Decision-Making Questions (%)	
	OB/GYNs	Pediatricians
Mechanisms of CMV lifecycle	75%	57%
CMV seropositivity rates among US adults	72%	81%
Duration of CVMV viral shedding	59%	56%
Source of CMV transmission	47%	34%
Strategies for diagnosing primary CMV	53%	58%
Prevalence of congenital CMV	79%	73%
Case-based diagnosis of CMV	72%	84%
Infant screening strategies for CMV	50%	42%
Strategies for prevention of primary CMV	35%	43%
Management of possible congenital CMV	74%	64%
Relationship between congenital CMV and congenital disabilities	67%	78%
Clinical presentation of an infant with congenital CMV	44%	35%
Treatment of congenital CMV in infant	60%	57%
Challenge in development of CMV vaccine	60%	56%
Current populations being studied for emerging CMV vaccines	72%	61%

